# Sesamin stimulates osteoblast differentiation through p38 and ERK1/2 MAPK signaling pathways

**DOI:** 10.1186/1472-6882-12-71

**Published:** 2012-05-30

**Authors:** Orawan Wanachewin, Kanchanit Boonmaleerat, Peraphan Pothacharoen, Vichai Reutrakul, Prachya Kongtawelert

**Affiliations:** 1 Thailand Excellence Center for Tissue Engineering and Stem Cells, Department of Biochemistry, Faculty of Medicine, Chiang Mai University, Chiang Mai 50200, Thailand; 2 Center for Innovation in Chemistry, Department of Chemistry, Faculty of Science, Mahidol University, Bangkok, Thailand

## Abstract

**Background:**

Osteoporosis is a worldwide health problem predominantly affecting post-menopausal women. Therapies aimed at increasing bone mass in osteoporetic patients lag behind comparable investigation of therapeutic strategies focusing on the bone resorption process. Sesamin, a major lignan compound found in *Sesamun indicum* Linn., has a variety of pharmacological effects, though its activity on bone cell function is unclear. Herein we examine the effect of this lignan on osteoblast differentiation and function.

**Method:**

Cell cytotoxicity and proliferative in hFOB1.19 were examined by MTT and alamar blue assay up to 96 h of treatment. Gene expression of *COL1, ALP, BMP-2, Runx2, OC, RANKL* and *OPG* were detected after 24 h of sesamin treatment. ALP activity was measured at day 7, 14 and 21 of cultured. For mineralized assay, ADSCs were cultured in the presence of osteogenic media supplement with or without sesamin for 21 days and then stained with Alizarin Red S. MAPK signaling pathway activation was observed by using western blotting.

**Results:**

Sesamin promoted the gene expression of *COL1*, *ALP*, *OCN*, *BMP-2* and *Runx2* in hFOB1.19. On the other hand, sesamin was able to up-regulate *OPG* and down-regulate *RANKL* gene expression. ALP activity also significantly increased after sesamin treatment. Interestingly, sesamin induced formation of mineralized nodules in adipose derived stem cells (ADSCs) as observed by Alizarin Red S staining; this implies that sesamin has anabolic effects both on progenitor and committed cell stages of osteoblasts. Western blotting data showed that sesamin activated phosphorylation of p38 and ERK1/2 in hFOB1.19.

**Conclusions:**

The data suggest that sesamin has the ability to trigger osteoblast differentiation by activation of the p38 and ERK MAPK signaling pathway and possibly indirectly regulate osteoclast development via the expression of *OPG* and *RANKL* in osteoblasts. Therefore, sesamin may be a promising phytochemical that could be developed for supplementation of osteoporotic therapy.

## Background

Osteoporosis, the most common metabolic bone disease, is characterized by low bone density and deterioration of bone micro-architecture [1]. This bone disease results from an imbalance in the bone remodeling process. Both a high rate of bone resorption and insufficiency of bone formation cause patients to develop bone fragility and possibly leading to bone fractures. The standard therapeutic drugs for osteoporosis include anti-resorptive drugs such as bisphosphonate, osteocalcin and estrogen, although these have little ability to stimulate new bone synthesis, which is important for patients with advanced bone loss [2-4]. Therefore, investigation of agents that improve bone formation is important as well.

Osteoblasts, or bone forming cells, are derived from mesenchymal stem cells (MSCs) that are also the progenitors of myocytes, chondrocytes and adipocytes [5]. Enhancement of osteoblast proliferation and differentiation can ameliorate both the quantity and quality of bone tissue. Osteoblast maturation and differentiation can be modulated through many kinds of environmental factors and signaling cascades [6-8]. Bone morphogenetic proteins (BMPs), a members of transforming growth factors (TGFs) are known to be essential for regulating osteoblast differentiation, especially via the Smad-dependent signaling pathway [9]. Meanwhile, cross-talk among other signaling pathways may also be involved in osteoblastogenesis.

Mitogen-activated protein kinase (MAPK) signaling occurs in many cells and involves in cell survival, proliferation and differentiation [10-13]. Many previous studies have shown that the expression of osteoblastogenic genes and functions are stimulated by MAPK signaling [14]. For example, the constitutively active form of ERK2 activates osteoblast differentiation both *in vitro* and *in vivo*[15,16] and p38 involves phosphorylation of smad-1 thus resulting in ALP expression and activation of osteoblasts [17].

Sesame seeds (*Sesamun indicum* L.) are widely used as dietary supplements. The plant is widely cultivated in Asian and African countries. The oil from the seed contains various phytochemical compounds that display medicinal properties. Jeng and Hou reported that health benefits of sesame seeds may be attributed to its lignans, especially sesamin [18]. Sesamin affects lipid metabolism, contributes to reduced incidence of tumorigenesis, and has the ability to protect neuronal cells against oxidative stress. The preventive ability of lignans on bone loss was reported [19], but effect on the bone formation process has as yet not been examined.

This study aimed to investigate sesamin’s effects on osteoblast differentiation by examination of osteoblastogenic related gene expression, ALP activity, the mineralization process, and an activation of p38 and ERK1/2 in the MAPK pathway. We also examined sesamin’s effect on *OPG/RANKL* gene expression, the important regulators of osteoclast differentiation.

## Methods

### Cell culture and treatment

Human fetal osteoblast cell line (hFOB1.19, CRL NO.11372) was purchased from ATCC and expanded in a 1:1 mixture of phenol red-free DMEM/Ham’s F-12 medium (Sigma-Aldrich) supplemented with 10% fetal bovine serum (FBS), 100 U/mL penicillin and 100 μg/mL streptomycin (basal media). Cells were incubated at a temperature of 33.5 °C with 95% air 5% CO_2_.

Human adipose derived stem cells (ADSCs) were purchased from Invitrogen. The cells were maintained in DMEM (Gibco) containing 10% FCS, 100 U/mL penicillin, 100 μg/mL streptomycin and were incubated at 37 °C with 95% air 5% CO_2_. The media was changed every three days. For osteogenic induction, additional components were 50 μg/ml L-ascorbic acid (Sigma-Aldrich), 10^-7^ M dexamethasone (Sigma-Aldrich) and 10 mM β-glycerophosphate (Fluka).

### Cell cytotoxicity assay

hFOB1.19 and ADSCs were plated in 96-well plates at a density of 5 x 10^3^ cells per well. Twenty-four hours after plating, the cells were exposed to 0.3-20 μg/ml sesamin for an additional 24, 48, 72 and 96 hours. Each treatment was carried out in triplicate. At the end of the treatment, 10 μl of MTT solution (0.5 mg/ml in PBS) was added to each well and the plate was incubated in a CO_2_ incubator at 37 °C for four hours prior to the buffer being decanted, and 100 μl DMSO was added to dissolve the formazan crystals. Optical density was measured at a wavelength of 540 nm using an ELISA plate reader. The percentage of cell viability was calculated by the equation:

(1) $$\%\text{of cell survival}=\left(\text{OD of sample}/\text{OD of control}\right)\text{x}100$$

### Cell proliferation assay

hFOB1.19 treatment were performed as described for the cytotoxic assay. At indicated time of treatment, alamar blue dye (10% v/v in culture medium) was added to each well and incubated again at 37 °C for four hours before the absorbance was measured at 540 nm (test wavelength) and 630 nm (reference wavelength) using a Titertek Multiskan M340 multiplate reader.

### RNA extraction and gene expression analysis

For examination of gene expression, hFOB1.19 was exposed to sesamin at 1.0, 2.5, 5.0 and 10.0 μg/ml for 24 hours. After that, the total RNA was extracted using Nucleospin® RNA II (Machere-Nagel) following the manufacturer’s instructions. The total RNA (2 μg) was reversibly transcribed to cDNA using the RevertAid^TM^ H First Strand cDNA Synthesis kit (Fermentas). Real-time quantitative polymerase chain reaction was performed in a DNA Engine (ABi 7500) using SYBR GREENER qPCR UNIVERSAL (Invitrogen), primer sequences as indicated in Table 1. Relative expression levels for each primer set were normalized to the expression of GAPDH by 2^-CT^ method [20].

**Table 1:** Primers used for real time-qPCR

**Gene**	**Sequence (5’-3’): Forward (F); Reverse (R)**	**Accession number**
*ALP*	F:CATGGCTTTGGGCAGAAGGA	NM_001114107.2
	R:CTAGCCCCAAAAAGAGTTGCAA	
*BMP-2*	F:ATGGATTCGTGGTGGAAGTC	NM_001200.2
	R:GTGGAGTTCAGATGTCAGC	
*Type I Collagen*	F:CAGCCGCTTCACCTACAGC	NM_000088.3
	R:TTTTGTATTCAATCACTGTCTTGCC	
*RANKL*	F: CACTATTAATGCCACCGAC	NM_033012.3
	R: GGGTATGAGAACTTGGGATT	
*Runx2*	F: GCCTTCAAGGTGGTAGCCC	NM_001024630.2
	R: CGTTACCCGCCATGACAGTA	
*OC*	F: GAAGCCCAGCGGTGCA	NM_199173.2
	R: CACTACCTCGCTGCCCTCC	
*OPG*	F: CCTCTCATCAGCTGTTGTGTG	NM_002546.3
	R: TATCTCAAGGTAGCGCCCTTC	
*GAPDH*	F:GAAGGTGAAGGTCGGAGTC	NM_002046.3
	R:GAAGATGGTGATGGGATTTC	

### Assessment of alkaline phosphatase activity

Cells were seeded in 24-well plates and treated with sesamin at 5 and 10 μg/ml up to 14 days. For all experiment periods, cells were maintained in osteogenic media, and media was changed every three days. ALP activity was detected using an alkaline phosphatase detection kit (Sigma-Aldrich). Briefly, the conditioned media were collected and 20 μl of media were incubated at 65 °C to diminish the background activity. Then samples were mixed with a reaction buffer, which contained 4-methylumbelliferyl phosphate disodium salt as substrate. Fluorescence at UV light wavelengths from 360/440 nm was measured with a multi-detection microplate fluorometer (Synergy ™ HT). The conditional medium of the day 3, 7 and 14 were measured for ALP activity and normalized with total protein level.

### Mineralization assay

ADSCs were stained with Alizarin Red S on day 21 of treatment for assessing the mineralized nodules. The medium was removed, and the cell layers were rinsed three times with PBS and fixed with 95% ethanol at room temperature for 15 min. Then, the cell layers were washed twice with deionized water. The fixed cells were stained with 2 ml of 40 mM Alizarin red-S (pH 4.0 - 4.5) per culture dish. After incubation at room temperature for 20 min, the cell layers were washed four times with an excess of deioinized water and observed under an inverted light microscope (Olympus BX41). For the quantitative method, stained cells were extracted and normalized with 10% acetic acid and 10% ammonium hydroxide respectively [21]. The absorbance of the solution was read at 405 nm in triplicate and calculated from the staining level using a standard curve.

### Western blotting

hFOB1.19 treated with sesamin for the indicated time and concentrations were lysed with a sample buffer containing 5% mercaptoethanol. For western blotting, the samples were subject to 12% gel SDS-PAGE. Then, the separated proteins were transferred to a nitrocellulose membrane (Amersham Pharmacia Biotech). The membrane was blocked for one hour with 5% skim milk in TBS and then incubated with specific primary antibody which are p38, ERK, JNK, phosphorylated p38, phosphorylated ERK, and phosphorylated JNK (Cell Signaling Technology) overnight at 4 ° C. After triplicate washing with TBS-Tween, the specific protein bands were probed with horseradish peroxide-labeled secondary antibody (Cell Signaling Technology) for one hour. Finally, the membranes were developed using an ECL kit (KPL).

### Statistical analysis

Data were expressed as means ± S.D. Statistically significant differences between the means of control and test group were assessed by independent *T*-test using SPSS software. p-value less than 0.05 (*) and 0.01 (**) were considered a significant difference.

## Results

### Sesamin did not affect cell viability and proliferation

**Figure 1:**
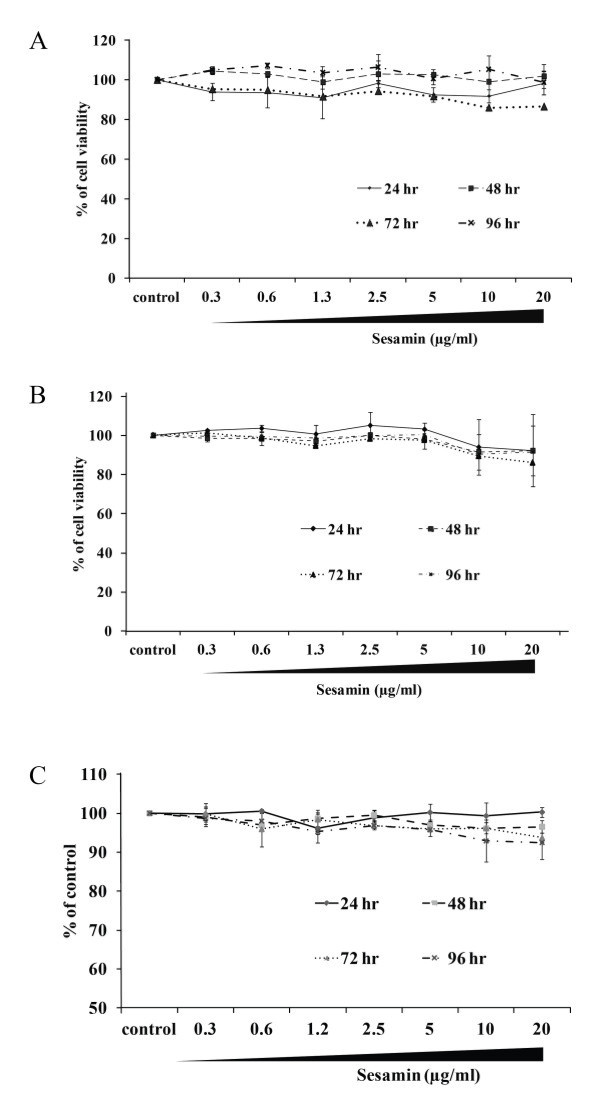
**The effects of sesamin on cytotoxicity of hFOB1.19 (A) ADSCs (B) and proliferation of hFOB1.19 (C) were examined at 24, 48, 72 and 96 h after sesamin treatment (0.3-20 μg/ml).** Data are shown as mean ± S.D. of three independent experimets. Statistical analysis was analyzed by independent *T*-test, p < 0.05.

Prior to the analysis of the anabolic effects of sesamin, the cytotoxicity on hFOB1.19 and ADSCs were investigated using the MTT assay, which is a reliable and widely used method of assessing cell viability based on mitochondrial enzyme activity [22]. After treatment with sesamin for 24, 48, 72 h, sesamin at various concentrations (0.3-20 μg/ml) had no cytotoxicity effect on hFOB1.19 and ADSCs, cells survival, as illustrated by the percentages of cellviability, which were more than 80% of those of the control group (Figure 1A and B). Besides, to examine whether sesamin had an effect on osteoblast proliferation, an alamar blue assay was performed. After 24, 48, 72 and 96 hours treatment, sesamin had no significant effect on the proliferation rate on human osteoblast cells (Figure 1C).

### Sesamin up-regulated osteoblastogenic genes

**Figure 2:**
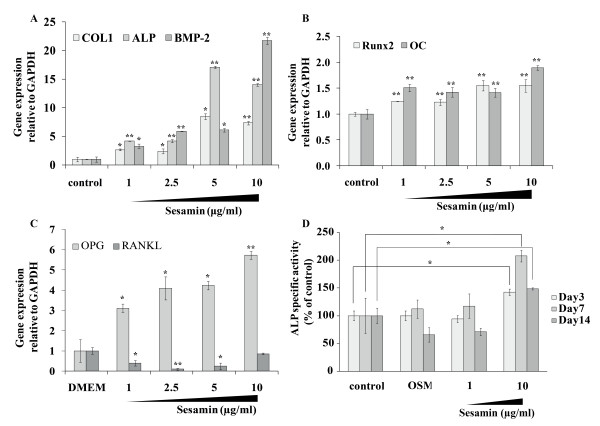
**Gene expression analysis and enzymatic activity of osteoblastogenic markers, for gene expression, 24 h FCS deprived hFOB1.19 cells were cultured in the presence of sesamin at indicated concentrations for another 24 h. cDNA of each samples was analyzed by Real-time PCR**. The expression of *COL1*, *ALP*, and *BMP-2* (**A**) The expression of *Runx2* and *OC*. (**B**) The expression of *OPG* and its counterpart *RANKL* (**C**) ALP activity was detected in culture media at days 3, 7 and 14 of culture using an alkaline phosphatase detection kit (**D**). All data are shown as mean ± S.D. of three independent experiments. Statistical analysis was analyzed by independent *T*-test, * and ** denoted for p-value ≤ 0.05 and 0.01, respectively.

During the bone formation process, there is increased expression of specific genes in osteoblasts; these genes play roles in extracellular matrix formation and mineral deposition. In order to study the anabolic effect of sesamin, we examined mRNA expression of *alkaline phosphatase* (*ALP*), *bone morphogenetic protein-2* (*BMP-2*), *runt related protein 2* (*Runx2*), *type I collagen*, and *osteocalcin (OC)*. *COL1*, *ALP* and *BMP-2* were highly up-regulated in the presence of sesamin. At the highest dose (10 μg/ml), expression levels of those genes were up to 7-fold, 15-fold and 20-fold increased relative to control, respectively (Figure 2A). Additionally, significantly increased expression of *Runx2* transcription factor and *osteocalcin* were observed in the culture with sesamin treatment (Figure 2B).

### Osteoclast differentiation may be regulated by sesamin via osteoblasts

Many studies have suggested that osteoclast differentiation is controlled by the action of the osteoblastic lineage through the RANKL/RANK/OPG system [23]. RANKL activates the development of pre-osteoclasts to become mature osteoclasts, while osteoprotegerin or OPG serves as a secreted receptor of RANKL (decoy receptor), resulting in inhibition of RANKL binding to its cell surface receptor and activation of osteoclast maturation. Because *RANKL* and *OPG* are both synthesized by the osteoblastic lineage, it is worthwhile to examine the expression levels of these genes under sesamin treatment. After 24 h of treatment, sesamin (2.5 and 5.0 μg/ml) up-regulated *OPG* gene expression, while the expression of *RANKL* was significantly decreased (Figure 2C). According to these results, sesamin might have an indirect inhibitory effect on osteoclasts via osteoblast function.

### ALP activity was stimulated by sesamin treatment

To verify the potential of sesamin on osteoblast differentiation, ALP activity was measured. Culture media were collected at days 3, 7, and 14 to measure ALP activity. Sesamin (10 μg/ml) significantly increased the enzyme activity at each date, while the highest stimulatory effect was observed on day seven, on which ALP activity levels were increased up to 208% compared with control. ALP activity of all treatments reached its highest levels on day 7 of the culture (Figure 2D). This result agreed with previous findings, that ALP is most highly expressed during the onset of the mineralization process, which lasts for about 7 days and decreases during later stages of bone formation [24].

### ADSCs were stimulated to form mineralized nodules by sesamin treatment

**Figure 3:**
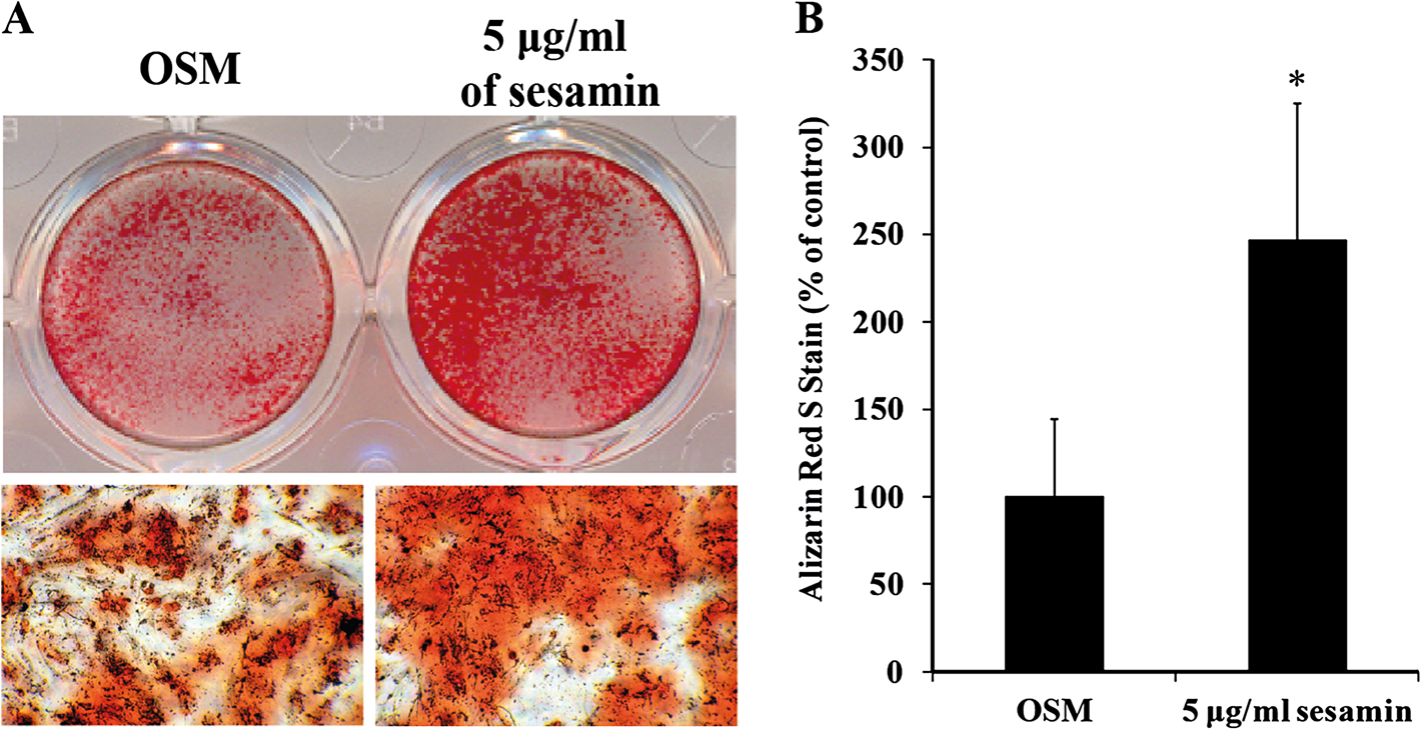
**Osteoblast differentiation of ADSCs was determined by Alizarin Red S staining.** Plate view (upper) and microscopic view (lower) of the staining on day 21 of sesamin treatment (**A**). Intensity of Alizarin Red S stains, which relative to quantity of mineralization process, were examined (**B**). Graph data are shown as mean ± S.D. of three independent experiments.

According to unobvious report of Alizarin Red S staining in hFOB1.19 shown by others [24,25] and undetectable of this stain performed in our sesamin treated hFOB1.19 that may be attributed to inadequate calcium supplement in recommend culture medium (DMEM/HAM F’12). Thus, the mineralization assay was performed in ADSCs, which are well studies about differentiation capacity as same as stem cells from other sources [26,27]. After 21 days of treatment, ADSCs had the potential to form mineralized nodules, which were observed as a bright red field with Alizarin Red S staining (Figure 3A). Quantification of Alizarin Red showed that sesamin significantly promoted mineral deposition by ADSCs, nearly up to 250% of the OSM control (Figure 3B). This suggests that sesamin can stimulate bone cell differentiation and bone matrix formation by ADSCs.

### Acceleration of osteoblast differentiation by sesamin might be attributed to p38 and ERK 1/2 MAPK signaling pathways

**Figure 4:**
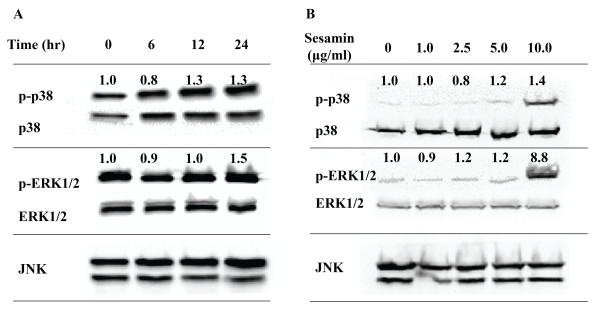
**MAPK signaling protein activation under vary times and concentration of sesamin treatment on hFOB1.19 by western blotting**. The numbers denoted the quality density value based on Quantity One 4.6.5 software. The MAPK signaling activation under vary time treatment (0–24 h) (**A**). The MAPK signaling activation under different concentration of sesamin treatment (**B**). The data is representative of three independent studies.

Although it is well documented that BMPs and Smad signaling pathways are enough to modulate bone cell growth and differentiation, cross-talking with other signaling pathways such mitogen activated kinase (MAPKs) are involved in bone formation. It has been reported that MAPK signaling mediate sesamin effects, therefore, we interested to test whether sesamin modulate osteoblast differentiation through MAPK signaling pathways. Sesamin treatment continuously increased phosphorylation of p38 and ERK in a time dependent manner (6, 12, 24 h). Band density analysis of signaling protein normalized to total form at 24 h treatment showed that sesamin (5.0 μg/ml) increased phosphorylated p38 and ERK1/2 up to 1.3 and 1.5 fold compared to control (Figure 4A). Concomitantly, 10.0 μg/ml sesamin treatment also significantly increased phosphorylated form of p38 and ERK1/2 (1.4 and 8.8 time compare to control) (Figure 4B), while, the activation of JNK was not found. To verify that JNK was not stimulated in this treatment condition, input p-JNK loaded on the same western blot was also determined as positive control. Phosphorylated JNK was detected only in input lane but not in cell lysate of sesamin treatments (data are not shown).

## Discussion

As mentioned concerning osteoporotic therapy, most drugs function as resorptive inhibitors. These types of drugs exhibit a negligible ability to enhance new bone synthesis.[2] Therefore, it is desirable to investigate potential anabolic agents that can correct the imbalance in the bone remodeling process. Phytochemical may be an effective addition to osteoporotic therapy. Sesamin, a major lignan component in sesame seeds, is one such promising compound, although to date there are no studies investigating the effect of sesamin on bone cell function. This report has demonstrated the efficiency of sesamin as an anabolic agent that can enhance osteoblast differentiation without effect on cell proliferation. The result showed that sesamin (at concentration 0.3-20 μg/ml) did not effects osteoblast cytotoxicity or proliferation as well as ADSCs viability. After 24 treatment in hFOB1.19, sesamin potentially up-regulated genes responsible for bone formation process which are *ALP, type I collagen* and *osteocalcin*. This rapid genes induction implied that sesamin may play role since early of osteoblast differentiation process. In addition, we found that *BMP-2* and *Runx2*, the well documented growth factor and transcription factor essential for osteoblast development [6,8,9,28,29], were up-regulated by sesamin on the hFOB1.19. This suggests that sesamin stimulate osteoblasts to be mature and function by accelerating osteoblastogenic gene expression.

The bone remodeling is well documented that mainly directed by two cell types, bone forming cells (osteoblasts) and bone removing cells (osteoclasts). Optimal functioning of these two cells is influenced by many environmental factors and signaling pathways [7,30]. We demonstrated that sesamin can activate p38 and ERK1/2 in MAPK pathway, which mediated osteoblast maturation and differentiation [15,16]. There is controversial evidence that MAPK signaling promotes osteoblast proliferation or differentiation [31]. Our study showed that elevation of p38 and ERK1/2 phosphorylated form is in accordance with elevated levels of gene expression essential for the mineralization process (*ALPtype I collagen* and *OCN*), which is in agreed with previous study that p-38 plays role in phosphorylation of smad-1 and smad-5 by BMP-2 stimulation [17]. Many studies reported the role of ERK in osteogenesis by enhance ALP activity in osteoblast progenitor cells [32-34]. Interestingly, sesamin up-regulated *Runx2* gene expression, in contrast to previous results reported by Xiao et al., that MAPKs can activate and phosphorylate Runx2 without affecting its expression level [35]. From our data, it may be that sesamin can stimulate osteoblast differentiation not only through p38/ERK activation but also via other signaling cascades, which should be further investigated.

ALP has been reported as a key enzyme for initiation of matrix deposition [36]. Since sesamin increased ALP activity, this may promote osteoblast differentiation. The sesamin treatment did not enhance the mineralizing effect in hFOB1.19, which might be due to a limitation on mineral deposition in this cell line. We thus performed additional experiments on ADSCs as potential sources of adult stem cells, that exhibits differentiate capacity into various of cell types [37]. Surprisingly, ADSCs can be induced by sesamin to deposit high quantity of mineralized nodules compared with control. The effect of sesamin on ADSCs implies that sesamin affected not only committed osteoblasts such hFOB, but also osteoprogenitors.

In addition to direct effects of sesamin on osteoblasts, there were indirect effects on osteoclast differentiation by up- regulating *OPG* expression and decreasing *RANKL* expression. During the bone remodeling process, there is communication among bone cells in order to modulate the homeogenesis of bone turnover [38]. The essential cross-talk, by which osteoblasts control differentiation of bone resorption cells or osteoclasts, is mediated through interaction between RANKL-expressing osteoblasts and RANK-expressing osteoclast precursors [23]. While RANKL binding leads to osteoclastogenesis activation, osteoblasts also express OPG, a decoy receptor of RANKL, leading to negatively controlled osteoclast development and further bone resorption. Treatment with sesamin at 2.5 and 5.0 μg/ml significantly increased the expression of *OPG* and decreased the expression of *RANKL*. These results suggest that sesamin might decrease the RANKL/OPG production ratio, resulting in indirect inhibition of osteoclastogenesis. Thus, regulation of *RANKL/OPG* expression may be one approach to reversing osteoporosis [2,39]. Nevertheless, sesamin (10.0 μg/ml) caused an elevated of *RANKL* gene expression. This inverse effect may be due to reciprocal regulation from its antagonist gene expression, *OPG*, to balance the expression ratio of both ligands.

Effects of sesamin on osteoblast differentiation as describe in this study is a good initiation highlight that phytochemical agent could be used as alternative therapy and prevention for bone loss disease. Since, osteoblast and osteoclast responsible for maintaining bone metabolism, thus, the effect of sesamin on osteoclast differentiation by direct and in-diract effects on osteoclast differentiation will be further investigated.

## Conclusions

Whereas the anti-inflammatory, anti-allergic and neuro-protective effects of sesamin are well documented, its effect on bone cells and associated diseases are relatively unknown. We examined the effects of sesamin on osteoblast differentiation and mineralization. Sesamin had direct effects on osteoblasts by stimulating the expression of essential genes and key enzymes of the bone mineralization process. This stimulation might occur due to the activation of p38 and ERK1/2 MAPK signaling pathways. Besides the direct effect on osteoblasts, sesamin may indirectly control osteoclast maturation and function through regulation of the expression ratio of *RANKL/OPG*. The effects of sesamin on *in vitro* bone differentiation in our study is in agreement with previous studies of Boulbaroud et al., who reported the preventive role of sesame oil in ovariectomized rats. Taken together, we concluded that sesamin is an effective candidate for bone disease therapy.

## Competing interest

The authors declare that they have no competing interests

## Authors’ contributions

OW carried out cell cytotoxicity assay, cell proliferation assay, mineralization assay, western blotting and drafted the manuscript. KB carried out Real-time RT-PCR, ALP activity and participated in study design and data analysis. PP supervised of the study protocol, data interpretation and manuscript preparation. VR and PK supervised the study design, data interpretation and corrected the manuscript for publication. All authors read and approved the final manuscript.

## Pre-publication history

The pre-publication history for this paper can be accessed here:

http://www.biomedcentral.com/1472-6882/12/71/prepub
